# Spatial memory deficiency early in 6xTg Alzheimer’s disease mouse model

**DOI:** 10.1038/s41598-020-79344-5

**Published:** 2021-01-14

**Authors:** Shinwoo Kang, Jinho Kim, Keun-A Chang

**Affiliations:** 1 Department of Pharmacology, College of Medicine, Gachon University, Incheon, 21999 Republic of Korea; 2 Neuroscience Research Institute, Gachon University, Incheon, 21565 Republic of Korea; 3 Department of Health Sciences and Technology, GAIHST, Gachon University, Incheon, 21999 Republic of Korea

**Keywords:** Molecular biology, Neuroscience, Molecular medicine

## Abstract

Alzheimer’s disease (AD) is mainly characterized by the deposition of extracellular amyloid plaques and intracellular accumulation of neurofibrillary tangles (NFTs). While the recent 5xFAD AD mouse model exhibits many AD-related phenotypes and a relatively early and aggressive amyloid β production, it does not show NFTs. Here, we developed and evaluated a novel AD mouse model (6xTg-AD, 6xTg) by crossbreeding 5xFAD mice with mice expressing mutant (P301L) tau protein (MAPT). Through behavioral and histopathological tests, we analyzed cognitive changes and neuropathology in 6xTg mice compared to their respective parental strains according to age. Spatial memory deficits occurred in 6xTg mice at 2 months of age, earlier than they occurred in 5xFAD mice. Histopathological data revealed aggressive Aβ42 and p-tau accumulation in 6xTg mice. Microglial activation occurred in the cortex and hippocampus of 6xTg mice beginning at 2 months. In 6xTg model mice, the synaptic loss was observed in the cortex from 4 months of age and in the hippocampus from 6 months of age, and neuronal loss appeared in the cortex from 4 months of age and in the hippocampus 6 months of age, earlier than it is observed in the 5xFAD and JNPL3 models. These results showed that each pathological symptom appeared much faster than in their parental animal models. In conclusion, these novel 6xTg-AD mice might be an advanced animal model for studying AD, representing a promising approach to developing effective therapy.

## Introduction

Alzheimer’s disease (AD) is progressive, neurodegenerative disease, and it is the most common form of dementia^1^. AD neuropathology is characterized by the deposition of extracellular amyloid plaques and the intracellular aggregation of neurofibrillary tangles (NFTs)^2–4^.

Several promising drugs have shown no clinical benefit in completed trials, and these disappointments led to a critical assessment of the value of relying only on AD’s rodent models before transitioning to human clinical trials^5^. For developing and evaluating effective treatment strategies for AD, many transgenic (Tg) mouse models representing the pathological features of AD have been developed^6^. Tg mouse models are largely based on the expression of specific genes with autosomal-dominant mutations of APP, PSEN1, and PSEN2 found in early-onset AD. Recently, the 5xFAD mouse expressing mutant APPswe/Ind/fl and PS1 with double FAD mutations (M146L and L286V) has been used in many preclinical studies. This model presents a robust age-dependent amyloid plaque, preceded by an increase in soluble Aβ40 and Aβ42, and an AD-related phenotype at an early age^7^. However, these Tg models do not show the other hallmark of AD—namely, NFT pathology—which is observed clearly in the human brain with AD.

A few studies have reported the development of animal models that show both plaques and tangles^8–12^. These models showing coexisting expression of mutated forms of APP, tau gene (MAPT), and occasionally also PSEN1 or PSEN2, display plaques and tangles in the same model. However, some problems have been reported in these models, owing to the consistent and abundant expression of both plaques and tangles. Also, the formation of both plaques and tangles in these models usually develops in old age. 5xFADxPS19 mice which crossed 5xFAD with Tau P301S (PS19) Tg mice showed a dramatically aggravated tauopathy with a dramatically increased inflammatory response and a loss of synapses and hippocampal CA1 neurons in aged mice and amyloidosis was unaltered^13,14^. 5xFADxTg30 mice made by crossing 5xFAD with Tau P301S/G272V (Tg30) Tg mice showed a more severe deficient motor phenotype than Tg30 mice, developed with age a dramatically accelerated NFT load in the brain compared to Tg30 mice, and amyloidopathy were lower than in 5xFAD mice^15^. The APP/PS1/rTg21221 line made by crossing APP/PS1 mice with rTg21221 mice overexpress WT human tau showed that accumulation of human wild‐type tau exacerbates plaque pathology and neurite deformation but not exacerbate plaque‐related synapse loss^16^. Although the 3xTg mouse model has been widely used in AD studies^17^, the widespread presence of plaques and tangles is typically not observed in these mice until old age, and even pathology is less than typically seen in AD^18^.

To develop a new AD animal model that more closely represents the pathological features of AD, we crossed 5xFAD mice with JNPL3 mice, the animal model of tauopathy expressing the P301L mutation. In JNPL3 Tg mice, NFTs and pretangles are widely distributed throughout the spinal cord and brain^3^. We then conducted a comparative analysis of the behavioral, histopathological, and neurological features in the novel 6xTg-AD mice and each parental line.

## Material and methods

### Generation of 6xTg-AD mice

We used 5xFAD (B6SJL) Tg mice (The Jackson Laboratory, ME, USA) that co-expresses human APP with the Swedish (KM670/671NL), Florida (I716V), and London (V717I) mutations, together with human mutant PS1 (M146L, L286V) driven by the mouse Thy1 promoter^7^. 5xFAD line was maintained by crossing hemizygous transgenic mice with B6SJL breeders (The Jackson Laboratory). We also used the JNPL3 Tg mouse (Taconic Biosciences Inc., NY, USA) that expresses human mutant tau (P301L) driven by the mouse prion promoter^3^. JNPL3 homozygous transgenic mice were maintained by breeding under a license agreement with Taconic Biosciences, Inc. For our study, we made JNPL3 hemizygous transgenic mice by breeding with B6SJL breeders (The Jackson Laboratory). For developing the 6xTg-AD (6xTg) mice, hemizygous 5xFAD transgenic mice were crossbred to JNPL3 hemizygous transgenic mice (B6SJL background), yielding animals with four different genotypes (wild type (W*T)*, JNPL3 ± (JNPL3), 5xFAD+/−(5xFAD), and JNPL3 ± 5xFAD+/−(6xTg); Fig. S1). Genotyping was performed on ear biopsy DNA by a polymerase chain reaction (PCR) with primers of PS1, APP, and MAPT. We performed further analysis of expression of the transgenes (APP, PSEN1, Tau) with brain tissues of 2-months-old mice in all groups by western blotting using 22C11 (against APP), PSEN1 (against PS1) and TAU (H150) (against total tau) antibodies (Figs. S1, S6).

All experiments were done blind with aspect to the genotype of mice and a series of analyses were carried out in order according to the schedule of Fig. S1 with age-matched male mice at 2, 4, 6, and 8 months of age. Animals were accommodated in an automatically controlled environment at 22 ± 2 °C and 50 ± 10% relative humidity under a 12-h light/dark cycle with ad libitum access to food and water. All procedures were conducted in accordance with the Animal Care and Use Guidelines of the Lee Gil Ya Cancer and Diabetes Institute, Gachon University, Incheon, Korea. And all experimental protocols were approved by the Institutional Animal Care and Use Committee of the Lee Gil Ya Cancer and Diabetes Institute, Gachon University (IACUC No. LCDI-2015–0024).

### Y-maze spontaneous alternation

Mice (n = 8–10; WT, JNPL3, 5xFAD, 6xTg at 2 months) were tested for the spontaneous alternation in a three-armed Y-maze constructed from white polyvinyl plastic. Each arm (A, B, and C) was 40-cm long, 6.8-cm wide, and 15.5-cm high and the three-folding angle was 120°^19^. Mice were placed in the equipment and allowed to explore freely. During each 8-min period, the number of times that the tail of each animal fully entered each arm was counted for each arm, and then, the number of times each animal entered the arm one after another (in A, B, or C sequence) was also counted, which was assigned one point (actual alternation). The inside of the Y-maze was wiped with 70% ethanol between different animal trials. Alternation behavior was defined as no overlap into all three arms and was calculated by the following equation: Rate of spontaneous alterations (%) = (number of alternations) / (the total number of arm entries − 2) × 100. All tests were recorded by a technician blind to the genotype of the animals^19^.

### Morris water maze (MWM) test

Spatial learning and memory were assessed using the MWM test using the Animal Cognitive Functions Assessment Meter (EthoVision Maze task system, Noldus Information Technology, Wageningen, Netherlands). Mice (n = 8–10; WT, JNPL3, 5xFAD, 6xTg at 2 months) were tested in a circular pool (90 cm diameter, 45 cm high, and outer height of 61.5 cm from the ground floor) filled with opaque water equilibrated to room temperature (22 °C). The tank was divided into four quadrants with different navigation marks (cues) for each quadrant. Mice were continuously trained with 4 trials per mouse each day (once per navigation mark) for 4 days to search for the escape platform within a maximum of 60 s^19^. During the test the platform location stayed constant and the time taken to reach the platform was recorded as the escape latency. After mouse found the hidden platform, it was kept on the platform for 5 s^20^. If mice could not find the platform within 60 s, they were placed on the platform for 20 s to encode the location of the escape platform; for these trials, the escape latency was recorded as 60 s. Mice removed from the pool were dried and returned to the home cage. The MWM probe test was performed within 48 h of the final trial. The platform was removed from the pool, and the mice were placed in the water and allowed to swim for 60 s. The time spent in the quadrant that previously contained the platform indicates long-term memory maintenance. Swim distance, velocity, and frequency were recorded as measures of motor function. All the tests were performed by a technician who was blind to the genotype of the animals.

### Collection of brain tissue

After the behavioral tests, the mice were terminated according to the experimental scheme (Fig. S1A). To collect brain tissue, mice were perfused transcardially with saline containing heparin under anesthesia with a mixture of Zoletil (8.3 mg/kg) and Rompun (15 mg/kg).

For 3,3′-Diaminobenzidine (DAB) immunohistochemistry, collected brains were fixed in 4% paraformaldehyde at 4 °C. After dehydration and paraffin-embedding, the paraffin-embedded tissues were cut into 4 μm-thick sections corresponding to the hippocampus regions using a microtome (Thermo Electron Corporation, MA, USA), and serial sections were placed on a glass slide^19^.

For fluorescent immunohistochemistry, fixed brains were additionally incubated in 30% sucrose solution at 4 °C for 72 h and then frozen. Frozen blocks of brain tissues were coronally cut into 30 μm-thick sections using a cryostat (Cryotome, Thermo Electron Corporation, Waltham, MA, USA), and stored at 4 °C in cryoprotectant solution (ethylene 30% and glycerol 30% in PBS)^21^.

For molecular studies, the cortical and hippocampal regions were dissected from the collected brain, and brain tissues were immediately frozen in liquid nitrogen. The frozen brain tissues were weighed and homogenized in RIPA buffer containing a protease inhibitor cocktail (Roche Applied Science, Mannheim, Germany) and a phosphatase inhibitors cocktail (Sigma-Aldrich)^19^. The lysates were incubated on ice for 30 min and then were centrifuged at 13,000 rpm and 4 °C for 10 min. The supernatants were collected for protein quantification using the Bio-Rad protein assay reagent, with bovine serum albumin as the standard.

### Immunohistochemistry

For immunohistochemistry on paraffin sections, 4 μm-thick coronal sections on a glass slide were deparaffinized and hydrated as described previously^19,22^. And then, the antigen retrieval process was done in 0.01 M citric acid at 56 °C, and non-specific binding of antibodies was blocked using PBST containing 5% normal goat serum three times for 5 min. Incubation with the primary antibody 6E10 (Sigma-Aldrich, St. Louis, MO, USA) and AT8 (Thermo Fisher Scientific, Tewksbury, MA, USA) in PBST containing 5% normal goat serum occurred overnight at 4 °C (Table 1). After three times washing with PBST for 5 min, the secondary biotinylated antibody (Vectastain Universal Elite ABC kit, Vector Laboratories, Burlingame, CA, USA) was applied at room temperature for 1 h and following three washes with PBS. The color reaction was developed by using the DAB substrate kit (Abcam). Counterstaining was carried out with hematoxylin. Sections were mounted with a coverslip using DAKO mounting medium (Agilent Tech., Santa Clara, CA, USA). Images were always taken from the same pre-defined region within the cortex and hippocampus using a Zeiss AxioImager Z1 microscope equipped with an Axiocam HRC camera (Carl-Zeiss Microscopy GmbH, Jena, Germany) and ImageJ software (v1.4.3.67, NIH, Bethesda, MD, USA). Serial images of × 200 magnification (prefrontal cortex, entorhinal cortex, hippocampus) were captured on 4 sections per animal. We counted every amyloid plaque or AT8-positive neuron (defined as “number of p-tau or PHFs”) in the images at 200 × magnification. The number of Aβ plaques and the amount of p-tau were quantified using an equal number of equally spaced sections with blind counting and presented as numbers in the area. The following animal models were used in these analyses: WT, JNPL3, 5xFAD, 6xTg (*n* = 5 each per time point).

**Table. 1.** List of antibodies used in this study.

Target	Species	Dilution	Company (Catalog)
***For DAB staining***			
Anti-6E10	Mouse	1:1000	Sigma-Aldrich (39,320)
Anti-AT8	Mouse	1:500	Thermo Fisher Scientific (MN1020)
***For Immunofluorescence staining***			
Anti-Iba1	Rabbit	1:300	Novus (NBP2-19,019)
Anti-MHC Class II	Mouse	1:200	Novus (NB100-65,541)
***For Western blot analysis***			
Anti-Synaptophysin	Mouse	1:3000	Abcam (ab8049)
Anti-NeuN	Rabbit	1:3000	Millipore (ABN78)
Anti-Iba1	Rabbit	1:3000	Novus (NBP2-19,019)
Anti-APP (22C11)	Mouse	1:3000	Millipore (MAB348)
Anti-PSEN1	Rabbit	1:2000	Cell signaling (CS5643)
Anti-Tau (H-150)	Rabbit	1:5000	Santa Cruz (SC5587)
Anti-GAPDH	Rabbit	1:5000	Bioworld (A531)
Anti-βactin	Mouse	1:3000	Santa Cruz (SC4777/8)
***Secondary Antibody***			
Goat Anti-Rabbit IgG-HRP Conjugate	1:8000~1:15,000		BIO-RAD (170–6515)
Goat Anti-Mouse IgG-HRP Conjugate	1:8000~1:15,000		BIO-RAD (170–6516)
Rabbit Anti-Goat IgG-HRP Conjugate	1:1:10,000		BIO-RAD (170–1034)
Alexa Fluor 555 Donkey anti rabbit IgG	1:1:500		Invitrogen (A-21429)
Alexa Fluor 555 Donkey anti mouse IgG	1:1:300		Invitrogen (A-21422)

For immunohistochemistry on frozen sections, the 30 μm-thick brain sections were washed three times in PBS containing 0.2% Triton X-100 and were then incubated in a blocking solution (0.5% bovine serum albumin and 3% normal goat serum in PBS with 0.4% Tween 20) for 1 h at RT^21^. The sections were incubated with the primary Iba-1 antibody (Novus Biologicals, Minneapolis, MN, USA) overnight at 4℃. Following this, the sections were washed three times and were then incubated with an Alexa Fluor 555 Donkey anti-rabbit IgG antibody (Invitrogen, Calsbad, CA, USA) for 1 h at RT. The brain slices were then washed three times and mounted onto slides using Antifade Mounting Medium with DAPI (Vector Laboratories, Burlingame, CA, USA). Tissue specimens were taken using a Nikon TS2-S-SM microscope equipped with a Nikon DS-Qi2 camera (Nikon Microscopy, Tokyo, Japan). Serial images of cortex and hippocampus were captured on 4 sections that were 30 μm afar from each other (× 100 magnification). Iba-1 stained brain slides of each group were compared and analyzed by region of interest (ROI) intensity ratio (%) using NIS-Elements software (BR 4.40.00, Nikon Microscopy). Once the ROIs were defined, it was used to measure the fluorescence intensity and percentage area of a red signal representing Alexa Fluro 555 within each ROI per section (3 mice per group)^23^.

### ELISA assay

The Aβ42, p-tau, and total tau levels in the soluble fraction of frozen brain tissues were quantified using an ELISA assay (Aβ42; Thermo Fisher Scientific, p-Tau; MyBioSource, San Diego, CA, USA, and total tau; Invitrogen)^23^. All assays were performed according to manufacturer instructions. Levels of these proteins were calculated from a standard curve developed with specific optical density versus serial dilutions of a known concentration. Each standard and experimental sample was run in duplicate, and the results were averaged^23^.

### Western blot analysis

The proteins in whole brain lysates was quantified using the BCA protein assay kit (Thermo Scientific) and separated by SDS-PAGE and transferred to a polyvinylidene difluoride (PVDF) membrane in transfer buffer^19^. The membrane was incubated for 1 h in blocking solution (6% skim milk) at room temperature, and incubated overnight with primary antibodies such as NeuN (Millipore, Billerica, MA, USA), synaptophysin (Abcam, Cambridge, UK), Iba1 (Novus, Littelton, CO, USA), APP (22C11, Milipore), PSEN1 (Cell signaling, Danver, MA, USA), Tau (H-150, Santa Cruz, Dallas, TX, USA), or GAPDH (Bioworld Technology, Inc., St. Louis Park, MN, USA) (Table 1)^19^. After being washed in Tris-buffered saline with Tween 20 (TBS-T) three times and incubated with a horseradish peroxidase-conjugated secondary antibody at room temperature for 1 h, the membrane was visualized using an Immobilon Western Chemiluminescent HRP Substrate (Millipore, WBKLS0500). The immunoblots were imaged using blue detection medical X-ray film (Agfa, Mortsel, Belgium) and densitometric analyses were performed by using ImageJ software (v1.4.3.67, NIH).

### Statistical analysis

All values are expressed as the mean ± SEM. All statistical analyses were performed using GraphPad Prism 8 software (GraphPad, La Jolla, CA, USA). For the Y-maze, data were analyzed using one-way analysis of variance (ANOVA) followed by the Tukey's multiple comparisons test. For the MWM training test, two-way repeated-measures ANOVA with Bonferroni multiple comparison corrections was used to compare the escape latency across 4 days of continuous hidden platform trials^19^. The MWM probe test was analyzed by one-way ANOVA followed by the Tukey's multiple comparisons test. In the IHC, ELISA, and WB assays, one-way ANOVA was used to examine group differences, followed by the Tukey's multiple comparisons test. A value of *p* < 0.05 was considered statistically significant for all measures.

## Results

### Cognition and memory impairment of 6xTg model mice

**Figure 1:**
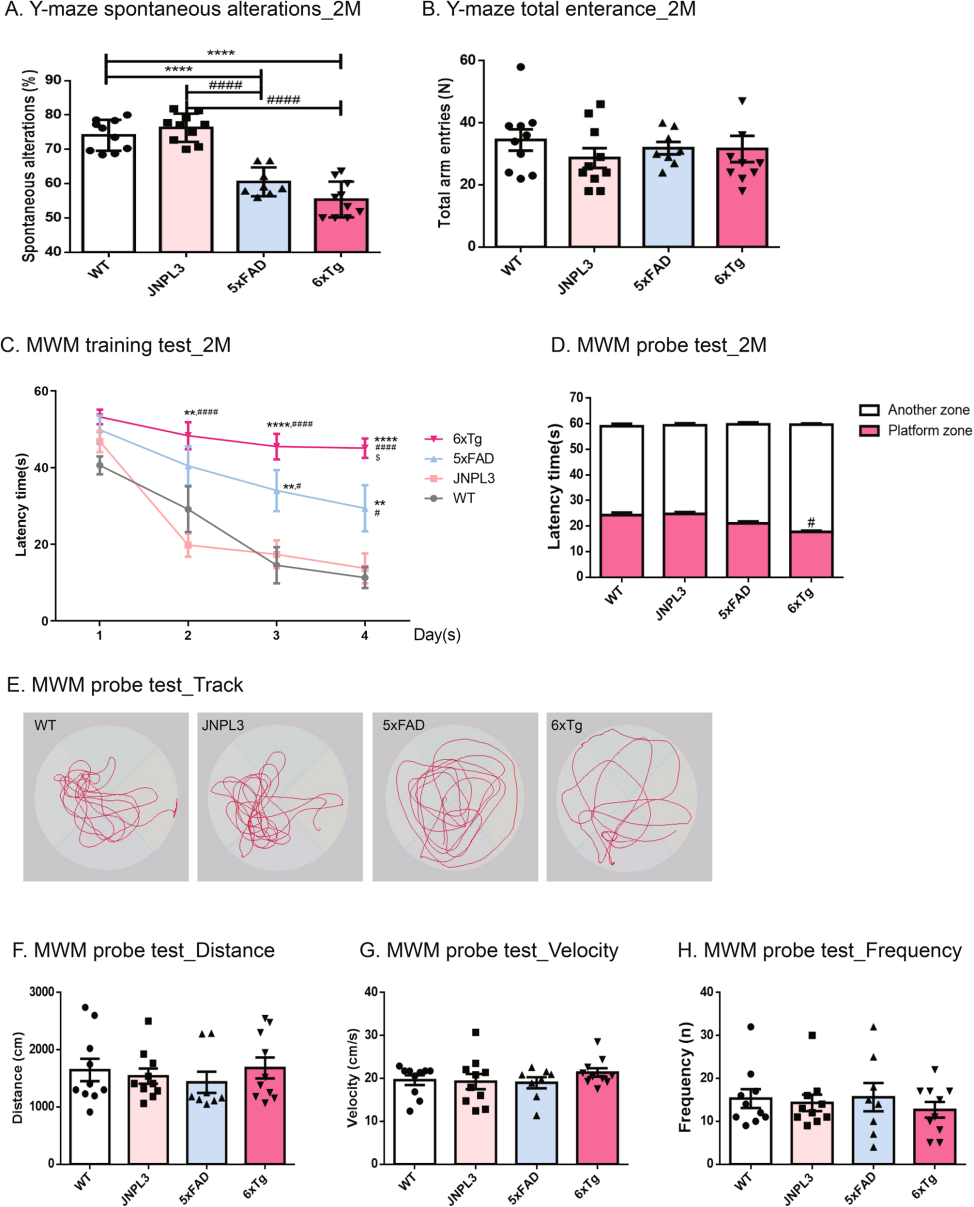
Learning and memory deficits in 2-month-old 6xTg mice. Two-month-old male 6xTg mice and their age- and gender-matched WT, JNPL3, and 5xFAD littermates were tested (n = 8–10 per group). (**A**, **B**) In the Y-maze test, 2-month-old male 6xTg mice showed significantly decreased spontaneous alternation as compared with WT mice. However, there was no significant difference in total entries across all groups. (**C**) In the acquisition training for the Morris water maze (MWM), escape latencies of all group mice decreased progressively over 4 days of training, regardless of the genotypes. However, 6xTg mice showed significantly increased latency time compared with WT mice. (**D**) In the probe test of MWM, 6xTg mice showed significantly reduced swimming time in the platform zone compared to the other groups. (**E**) Representative swim paths during the probe test. We did not observe any difference in distance (**F**), velocity (swim speed) (**G**), or frequency (**H**) during the probe test. All data are given as means ± SEM. The statistical analyses were performed by one-way ANOVA followed by the Tukey's multiple comparisons test, and for MWM, a two-way repeated-measures ANOVA followed by a Bonferroni multiple comparisons correction was used to compare the escape latency in 4 days of continuous hidden platform trials. ****p < 0.0001, **p < 0.01 vs. WT; ^####^*p* < 0.0001, ^##^*p* < 0.05 vs. JNPL3; $*p* < 0.01 vs. 5xFAD.

To evaluate the cognitive impairment of 6xTg-AD model (6xTg) mice, we performed three kinds of behavioral tests of 4 groups (wild type [W*T]*, JNPL3, 5xFAD, 6xTg) of mice aged 2 months. First, in the Y-maze test, all groups of mice aged 2 months were evaluated for short-term memory and procedural working memory. The 2-month-old 6xTg mice (55.40 ± 1.65, *p* < 0.0001 vs. WT, *p* < 0.0001 vs. JNPL3) displayed a significant decrease in spontaneous alteration changes compared with WT mice (74.10 ± 1.43), JNPL3 mice (76.29 ± 1.30) or 5xFAD mice (60.53 ± 1.48) (Fig. 1A). The total number of arm entries was not markedly different among the groups (Fig. 1B). In the MWM, all groups of 2-month-old mice were evaluated for spatial reference and working memory associated with the hippocampus. At the last day of an acquisition training test, 2-month-old 6xTg mice (45.09 ± 2.52, *p* < 0.0001 vs. WT, *p* < 0.0001 vs. JNPL3, *p* < 0.05 vs. 5xFAD) exhibited a significantly increased escape latency time relative to WT mice (11.33 ± 2.76), JNPL3 mice (13.75 ± 3.89), and 5xFAD mice (29.41 ± 6.02, *p* < 0.01 vs. WT, *p* < 0.05 vs. JNPL3) (Fig. 1C). In the probe test, the 6xTg mice (17.78 ± 0.47) displayed a decrease in the time spent in the platform zone compared with WT mice (24.28 ± 1.02, ns), JNPL3 mice (24.79 ± 0.70, *p* < 0.05), or 5xFAD Tg (21.12 ± 2.07, ns), and in particular, showed a significant difference compared to JNPL3 mice (Fig. 1D,E). We did not observe any difference in distance (Fig. 1F), velocity (swim speed; Fig. 1G), or frequency (Fig. 1H) among the groups during the probe test. These results show that 6xTg mice have markedly decreased cognitive and memory capacity at 2 months compared with WT, JNPL3, or 5xFAD mice.

### Amyloid deposition in 6xTg mouse brain

**Figure 2:**
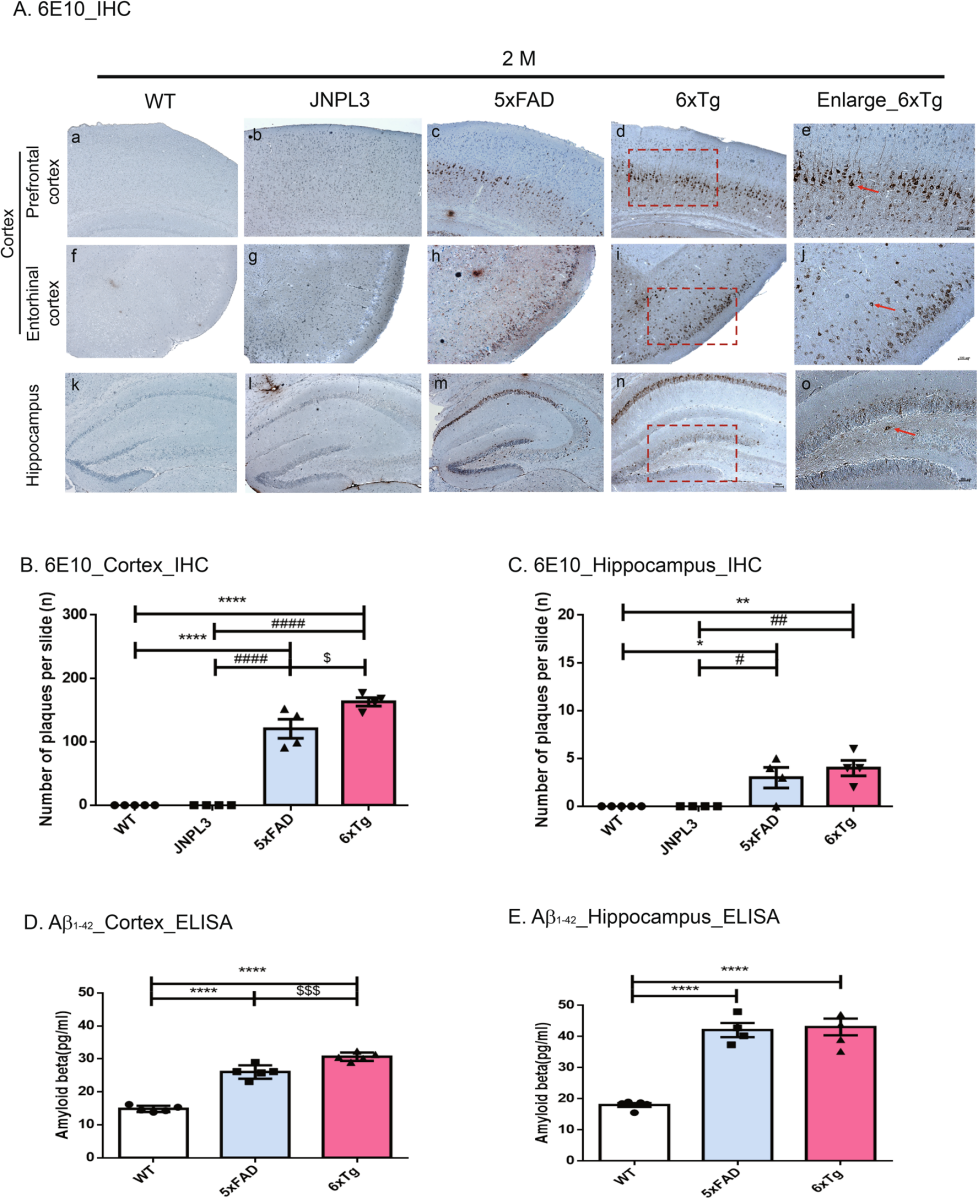
Age-dependent increase of Aβ plaques and amount of Aβ in the brains of 6xTg mice. (**A**) Immunohistological staining of amyloid plaques and tau phosphorylation with anti-Aß (6E10) in the cortex [prefrontal cortex (**a**–**d**) and entorhinal cortex (**f**–**i**)] and hippocampal dentate gyrus (k–n) of 2-month-old 6xTg mice and their age- and gender-matched WT, 5xFAD, and 6xTg littermates. Scale bar 200 μm. The red box contains enlarged images of cortical and hippocampal regions of 6xTg mice (e, j, o; Scale bar 100 μm). Red arrow-shaped 6E10-positive neurons were counted. (**B**, **C**) In the 6xTg mice, the number of plaques was significantly increased compared with WT mice, but Aß deposition showed no significant difference compared with the 5xFAD mice, but showed a tendency to increase in the hippocampus. (**D**, **E**) Aß proteins from cortical and hippocampal brain lysates of mice were subjected to ELISA assay and levels of Aß were examined. All data are given as means ± SEM (n = 5 mice per group). Statistical analyses were performed by one-way ANOVA followed by the Tukey's multiple comparisons test. ****p < 0.0001, ***p < 0.001, **p < 0.01 vs, *p < 0.05 vs. WT; ^####^*p* < 0.0001, ^###^*p* < 0.001, ^##^*p* < 0.01, ^#^*p* < 0.05 vs. JNPL3; ^$$$^*p* < 0.001 vs, ^$^*p* < 0.05 vs. 5xFAD.

To directly assess the actual Aβ burden in the brain of 6xTg mice, we performed immunohistochemistry with a specific antibody against Aβ (6E10 antibody) in 2-, 4-, 6-, and 8-month-old male WT, JNPL3, 5xFAD, and 6xTg mice. At 2 months old, Aβ deposition appeared only in the 5xFAD and 6xTg mice, and the amount of Aβ deposition had aggressively increased by 8 months (Fig. S2A,B&C). As shown in Fig. 2A, Aβ deposition was significantly increased in the 2-month-old 6xTg mice compared with the 5xFAD mice. In the cortex and hippocampus, significantly more plaques were observed in the 6xTg mice (cortex, 163.25 ± 6.52; hippocampus, 4.00 ± 0.82) than in the 5xFAD mice (cortex, 120.80 ± 15.12, *p* < 0.01; hippocampus, 3.00 ± 1.08, *p* < 0.01) (Fig. 2B,C). Using Thioflavin-S (Thio-S) staining, amyloid plaques were confirmed in the brains of 5xFAD and 6xTg mice, but, no plaque was found in the brains of WT & JNPL3 mice (Fig. S8A). Aβ deposition was significantly increased in the subiculum of the 2-month-old 6xTg mice (29.33 ± 0.98, *p* < 0.001) compared with the 5xFAD mice (12.67 ± 2.76) (Fig. S8B). At 4 months old, more plaques were observed in the subiculum of 6xTg mice (41.67 ± 2.333) than in the subiculum of 5xFAD mice (34.00 ± 3.786) (Fig. S8C).

To verify the level of amyloid proteins in the brains, we performed an ELISA assay for detecting amyloid β1-42 in the collected brain tissue supernatants from WT, 5xFAD, and 6xTg mice. There were dramatic differences among WT, 5xFAD, and 6xTg mice. Amyloid β1-42 levels in the cortex of 6xTg mice (30.67 ± 0.56) were significantly higher than those in WT (14.84 ± 0.41, *p* < 0.0001) and 5xFAD (26.02 ± 0.91, *p* < 0.001) mice at 2 months (Fig. 2D). In the hippocampus, 2-month-old 6xTg mice (43.07 ± 2.69) presented a greater amount of amyloid β1-42 relative to age-matched WT (18.03 ± 0.63, *p* < 0.0001) and 5xFAD (42.09 ± 2.26, *ns*) mice (Fig. 2E).

### Tau deposition in 6xTg mouse brains

**Figure 3:**
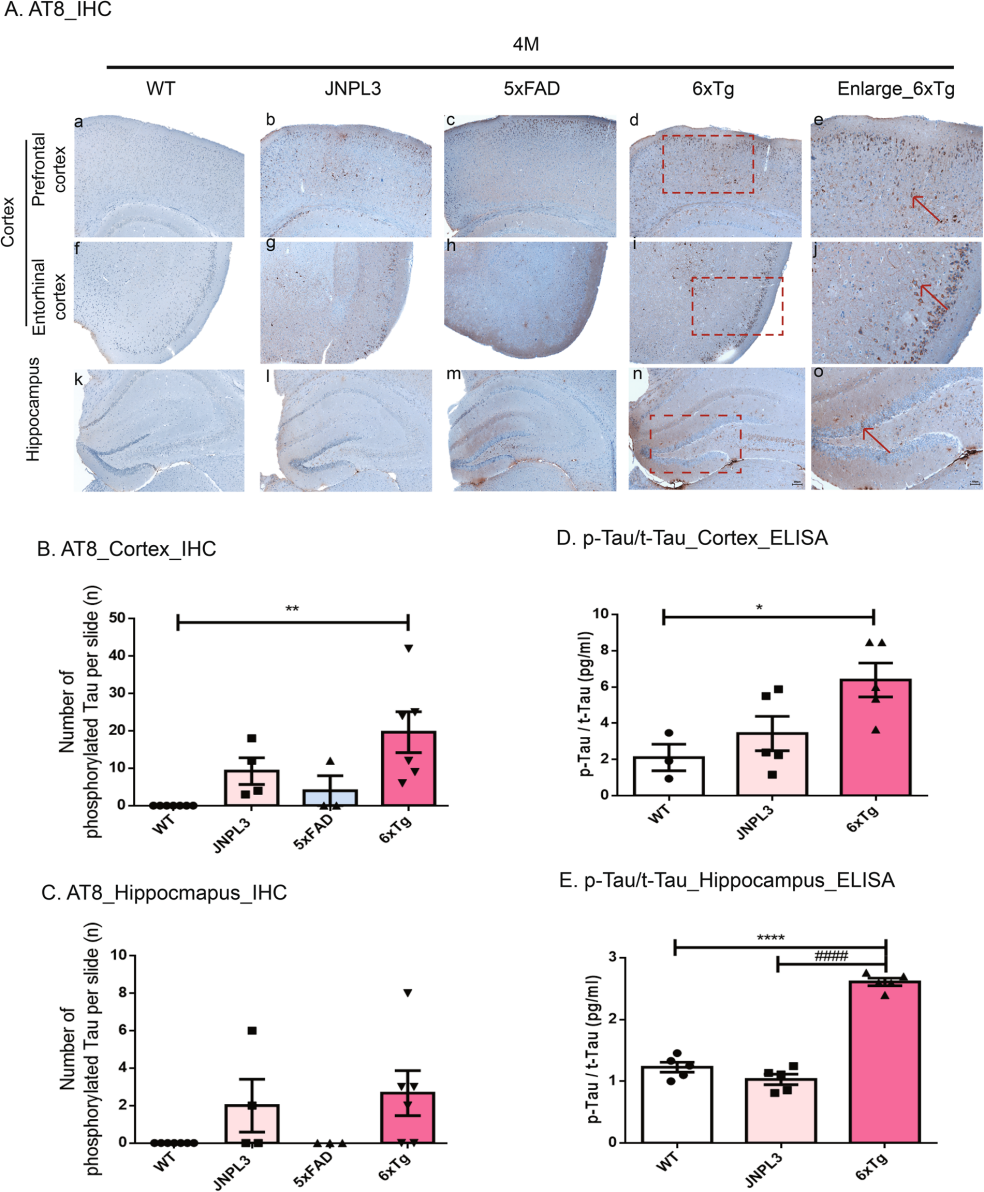
Age-dependent increase of PHF tau deposits and phosphorylated tau amounts in the brains of 6xTg mice. (**A**) Immunohistological staining of tau pathology with anti-phosphorylated Tau (AT8) in the cortex [prefrontal cortex (**A**–**D**) and entorhinal cortex (**F**–**I**), CX] and hippocampal dentate gyrus (K–N, HP) of 4-month-old 6xTg mice and their age- and gender-matched WT, JNPL3 or 6xTg littermates. The red box contains enlarged images of the cortical and hippocampal regions of 6xTg mice (**E**, **J**, **O**). Red arrow-shaped AT8-positive neurons were counted. (**B**, **C**) In the 4-month-old mice, PHF tau deposits in the cortex of 6xTg mice showed a significant increase compared with 5xFAD and WT mice, but the number of phosphorylated tau in the hippocampus of 6xTg mice showed no significant difference compared with 5xFAD mice, but showed a tendency to increase. (**D**, **E**) Phosphorylated tau (p-Tau) and total tau (t-tau) from cortical and hippocampal brain lysates of mice were subjected to ELISA assay and levels of p-Tau/t-tau were examined. All data are given as means ± SEM (n = 3–5 mice per group). Statistical analyses were performed by one-way ANOVA followed by the Tukey's multiple comparisons test. ****p < 0.0001, ***p < 0.001, **p < 0.01 vs, *p < 0.05 vs. WT; ^##^*p* < 0.01 vs. JNPL3; ^$$$$^*p* < 0.0001 vs, ^$$^*p* < 0.01 vs. 5xFAD.

To investigate whether transgenic overexpression of mutant APP influences tau pathology in JNPL3 mice, we performed immunohistochemistry with the AT8 specific p-tau antibody in the 2- 4-, 6-, and 8-month-old male of WT, JNPL3, 5xFAD, and 6xTg mice. P-tau is the main component of the paired helical filaments (PHFs) in AD. As shown in Fig. S3, PHF tau was detected in the brain tissue of 4-month-old JNPL3, 5xFAD, and 6xTg mice, but not WT mice, and the amount had progressively increased by 8 months (Fig. S3A). As expected, 6xTg mice showed a marked increase in the amount of PHF tau in the cortex and hippocampus, like parental JNPL3 mice (Fig. S3B&C). PHF tau deposition was significantly increased in 4-month-old 6xTg mice (19.67 ± 5.01) compared with the 5xFAD mice (4.00 ± 3.27, *p* < 0.01) in the cortex (Fig. 3B). There was a tendency toward an increase in the cortex of 4-month-old 6xTg (19.67 ± 5.01) mice compared to JNPL3 (9.25 ± 3.07, ns), which did not reach statistical significance (Fig. 3B). However, 6-month-old 6xTg mice (26.25 ± 1.82) showed a significant increase of PHF tau in the cortex as compared with the JNPL3 mice (12.75 ± 1.52,* p* < 0.001) (Fig. S3B). In the hippocampus, the 6xTg mice (2.67 ± 1.20) had a higher amount of PHF tau than did the 5xFAD mice (0.00 ± 0.00, *ns*) or JNPL3 (2.00 ± 1.41), but it was not significant (Fig. 3A,C). 8-month-old 6xTg mice (3.14 ± 0.96) showed a significant increase of PHF tau in the hippocampus as compared with the 5xFAD mice (0.33 ± 0.33, *ns*) (Fig. S3C).

To confirm changes of p-tau and total (t) tau protein levels in the brain, an ELISA assay was performed. As shown in Fig. 3D,E, p-tau/t-tau level was significantly increased in the cortex of 6xTg mice (3.02 ± 0.41) compared with WT (1.00 ± 0.34, *p* < 0.05) or JNPL3 (1.62 ± 0.44, *ns*) mice at 4 months (Fig. 3D). P-tau/t-tau level (2.53 ± 0.11) in the hippocampus of 4-month old 6xTg mice were significantly increased compared with WT (1.00 ± 0.08, *p* < 0.0001) and a tendency toward an increased p-tau/t-tau level was observed compared with their JNPL3 littermates (2.64 ± 0.19, ns), which did not reach statistical significance.

### Microglia activation in 6xTg mice

Amyloid and tau pathologies are known to cause neuroinflammation^24^. To investigate the microglia activation in the brains of 6xTg mice, we performed immunohistochemistry and western blot with the Iba-1 antibody in the 2- and 4-month-old male of WT, JNPL3, 5xFAD, and 6xTg mice.

**Figure 4:**
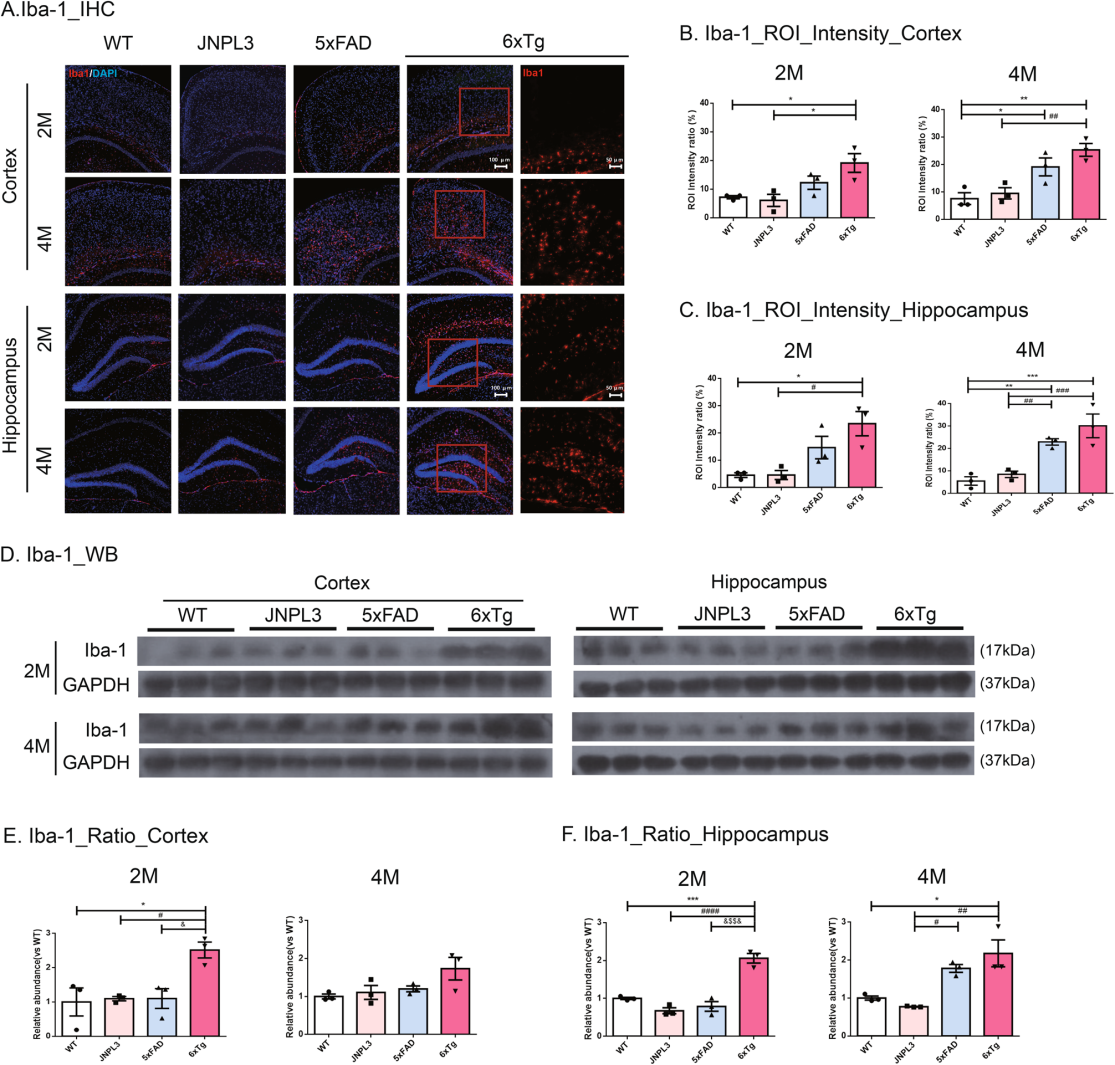
Activated microglia in the cortex and hippocampus of 6xTg mice brains. Activated microglia were identified in the brains of 2- and 4-month-old WT, JNPL3, 5xFAD, and 6xTg mice by immunofluorescent staining and Western blot analysis with an anti-Iba1 antibody. (**A**) Representative Iba-1-immunofluorescent images (red) in the brains of 2- and 4-month-old WT, JNPL3, 5xFAD, and 6xTg mice. The sections were counterstained with DAPI (blue). The upper panels are cortical regions (cortex), and the lower panels are hippocampal regions (hippocampus). (Scale bar, 100 μm). The right panels are enlarged images of the brain sections of 6xTg mice at 2 and 4 months of age. (Scale bar, 50 μm). Immunofluorescent staining showed that activated microglial cells were increased in the cortical and hippocampal areas of 2- and 4-month-old 6xTg mice brains compared to WT and JNPL3 mice. Quantification analysis of activated microglia in the cortex (**B**) and hippocampus (**C**) of 2- and 4-month-old WT and 6xTg mice is represented as an ROI intensity ratio (%). All data were given as means ± SEM (n = 3 mice per group). Statistical analyses performed by one-way ANOVA followed by the Tukey's multiple comparisons test. ***p < 0.001, **p < 0.01, *p < 0.05 vs. WT. ^###^*p* < 0.001, ^##^*p* < 0.01, ^#^*p* < 0.05 vs. JNPL3. (**D**) Representative Western blots of Iba-1 in the cortex and hippocampus were shown. In Fig. S4, full-length blots were presented. (**E**, **F**) The graph shows the percentage of the density of Iba-1 normalized to GAPDH on Western blot bands from cortical and hippocampal tissue lysates. In the 2-month-old mice, the Iba-1 proteins of the 6xTg mice were significantly increased compared with the 5xFAD mice as well as WT and JNPL3 mice. All data are shown as means ± SEM, and each experiment was repeated five times (n = 3 per group). Statistical analyses were performed by one-way ANOVA followed by the Tukey's multiple comparisons test. ***p < 0.001, *p < 0.05 vs. WT; ^####^*p* < 0.0001, ^##^*p* < 0.01, ^#^*p* < 0.05 vs. JNPL3; ^$$$$^*p* < 0.0001, ^$^*p* < 0.05 vs. 5xFAD.

Microglial activation was greater in the cortex and hippocampus of 6xTg mice at 4 months of age (Fig. 4A) than it was at 2 months (Fig. 4A). The ROI intensity ratios of Iba-1-positive microglia in the cortex and hippocampus were measured in 2- and 4-month-old WT and 6xTg mice (Fig. 4B,C). In the cortex, the ROI intensity ratio increased progressively with age in 6xTg mice, but not in WT mice (Fig. 4B). In 2-month-old 6xTg mice, a tendency toward an increased Iba-1 level was observed compared with their JNPL3 or 5xFAD littermates. Notably, we observed that Iba-1 positive brain cells were significantly increased in the cortex of 6xTg (18.62 ± 1.96) compared with WT (7.18 ± 0.54, *p* < 0.05) and JNPL3 (6.07 ± 2.22, *p* < 0.05) mice (Fig. 4B,C). Even in the hippocampus, Iba-1 positive brain cells of 6xTg (23.44 ± 4.46) were significantly increased compared to WT (4.53 ± 0.84, *p* < 0.05) and JNPL3 (4.57 ± 1.66, *p* < 0.05). However, there was an increasing trend in 6xTg compared to 5xFAD (cortex, 12.24 ± 2.3; hippocampus, 14.66 ± 4.13), but there was no significant difference (Fig. 4B,C). At 4 months of age, strong Iba1 immunoreactivity was shown in the cortex and hippocampus of 6xTg mice (Fig. 4C). Immunoreactivity in 4-month-old 6xTg mice (cortex, 25.32 ± 2.33) was higher than that of age-matched WT (7.53 ± 2.16, *p* < 0.01), JNPL3 (9.45 ± 2.05, *p* < 0.01), or 5xFAD (19.11 ± 3.27, ns) mice (Fig. 4B,C). In the hippocampus of 4-month-old mice, the Iba-1 intensity of 6xTg (30.04 ± 5.28) was higher than that of age-matched WT (5.47 ± 1.90, *p* < 0.001), JNPL3 (8.44 ± 1.44, *p* < 0.01), or 5xFAD (22.87 ± 1.44, ns) mice (Fig. 4B,C).

Next, we tried to confirm the increased amount of Iba-1 in the brain of 6xTg mice using western blot (Fig. 4D). The expression levels of Iba-1 in the cortex were increased in 2-month-old 6xTg mice (2.51 ± 0.18) compared to WT (1.00 ± 0.33, *p* < 0.05), JNPL3 (1.09 ± 0.05, *p* < 0.05), and 5xFAD (1.10 ± 0.23, *p* < 0.05) mice (Fig. 4E). In the hippocampus, the expression levels of Iba-1 were increased in 2-month-old 6xTg mice (2.06 ± 0.01) compared to WT (1.00 ± 0.02, *p* < 0.001), JNPL3 (0.67 ± 0.06,* p* < 0.0001), and 5xFAD (0.79 ± 0.10,* p* < 0.0001) mice (Fig. 4F). The activation of microglia in 6xTg mice was confirmed using anti-MHC Class II antibody (Fig. S7A). MHC Class II-positive cells were observed to increase with age in the cortex (CX) and dentate gyrus of the hippocampus (HP) of the 2- and 4-month-old 6xTg mice (cortex, 22.00 ± 2.08; hippocampus, 12.67 ± 1.45 at 2 months) (cortex, 33.67 ± 4.91; hippocampus, 20.00 ± 3.79 at 4 months), but no MHC Class II staining was seen in WT & JNPL3 mice (Fig. S7B&D).

### Neuronal and synaptic loss in 6xTg mice

**Figure 5:**
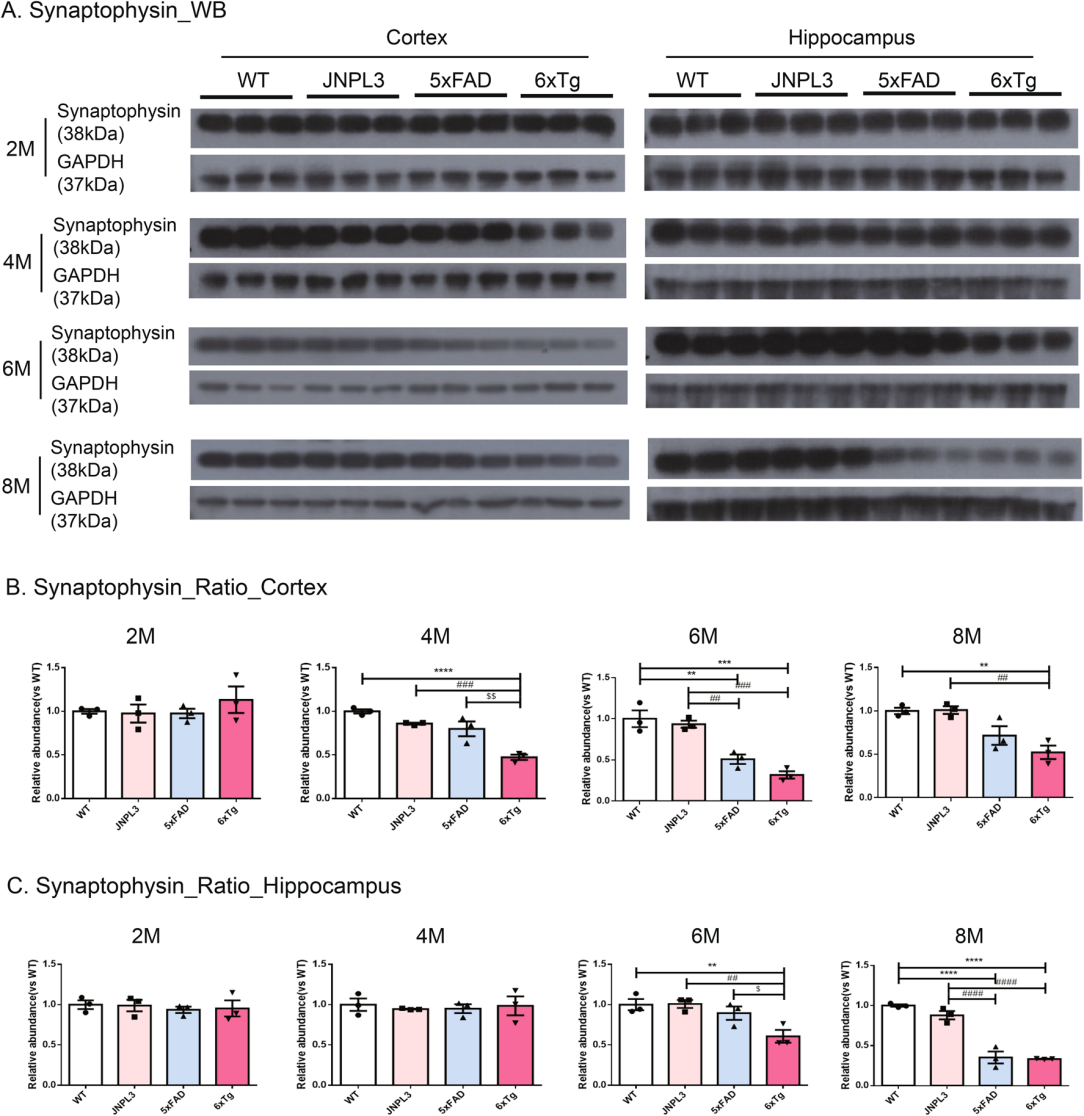
Synaptophysin levels in the cortical and hippocampal area of 6xTg mice brains. Presynaptic receptor proteins (Synaptophysin, 38 kDa) were detected in cortical and hippocampal brain lysates of 2-, 4-, 6-, and 8-month-old WT, JNPL3, 5xFAD, and 6xTg mice by Western blot analysis. (**A**) Representative Western blots of synaptophysin from cortical and hippocampal brain lysates were shown. In Fig. S5, full-length blots were presented. (**B**, **C**) The graph shows the percentage of the density of synaptophysin normalized to GAPDH on Western blot bands from cortical and hippocampal tissue lysates. (**B**) In the cortex, 4-, 6- and 8-month-old 6xTg mice showed significantly decreased synaptophysin compared with 5xFAD and WT mice. (**C**) In the hippocampus, 6- and 8-month-old 6xTg mice showed significantly decreased synaptophysin compared with 5xFAD and WT mice. All data are shown as means ± SEM, and each experiment was repeated five times (n = 3 per group). Statistical analyses were performed by one-way ANOVA followed by the Tukey's multiple comparisons test. ****p < 0.0001, ***p < 0.001, **p < 0.01 vs. WT; ^####^*p* < 0.0001, ^###^*p* < 0.001, ^##^*p* < 0.01, ^#^*p* < 0.05 vs. JNPL3; ^$$^*p* < 0.01, ^$^*p* < 0.05 vs. 5xFAD.

From the findings, it seems that the age-dependent formation of the neuritic plaque and tau deposition induced synaptic reduction and neuronal loss. To determine the loss of synapse number on the integrity of synapses, we examined the levels of synaptophysin proteins (Fig. 5A). At 2 months of age, there was no significant difference in the synaptophysin amount in the cortex and hippocampus among WT, 5xFAD, and 6xTg mice. In the cortex of 5xFAD mice a significant decrease of synaptophysin levels was shown from 6 months of age compared to WT and JNPL3 mice (5xFAD ratio vs WT; 0.67 ± 0.04, *p* < 0.01 vs. WT, *p* < 0.05 vs. JNPL3) (Fig. 5B). At 8 months of age, the amount of synaptophysin content was significantly reduced in both the cortex and hippocampus of 5xFAD (Fig. 5B,C). However, in the cortex of 4-months-old 6xTg mice, the synaptophysin amount was significantly decreased relative to age-matched WT, JNPL3, or 5xFAD mice (6xTg ratio vs WT; 0.47 ± 0.02, *p* < 0.001 vs. WT, *p* < 0.001 vs. JNPL3, *p* < 0.01 vs. 5xFAD) (Fig. 5B). At 6 months of age, the quantity of synaptophysin was significantly decreased in both the cortex and hippocampus of the 6xTg mice compared to WT, JNPL3, and 5xFAD mice (cortex: 0.46 ± 0.02, *p* < 0.001 vs. WT, *p* < 0.05 vs. JNPL3; hippocampus: 0.60 ± 0.06, *p* < 0.01 vs. WT, *p* < 0.05 vs. JNPL3*, p* < 0.01 vs. 5xFAD) (Fig. 5B,C).

**Figure 6:**
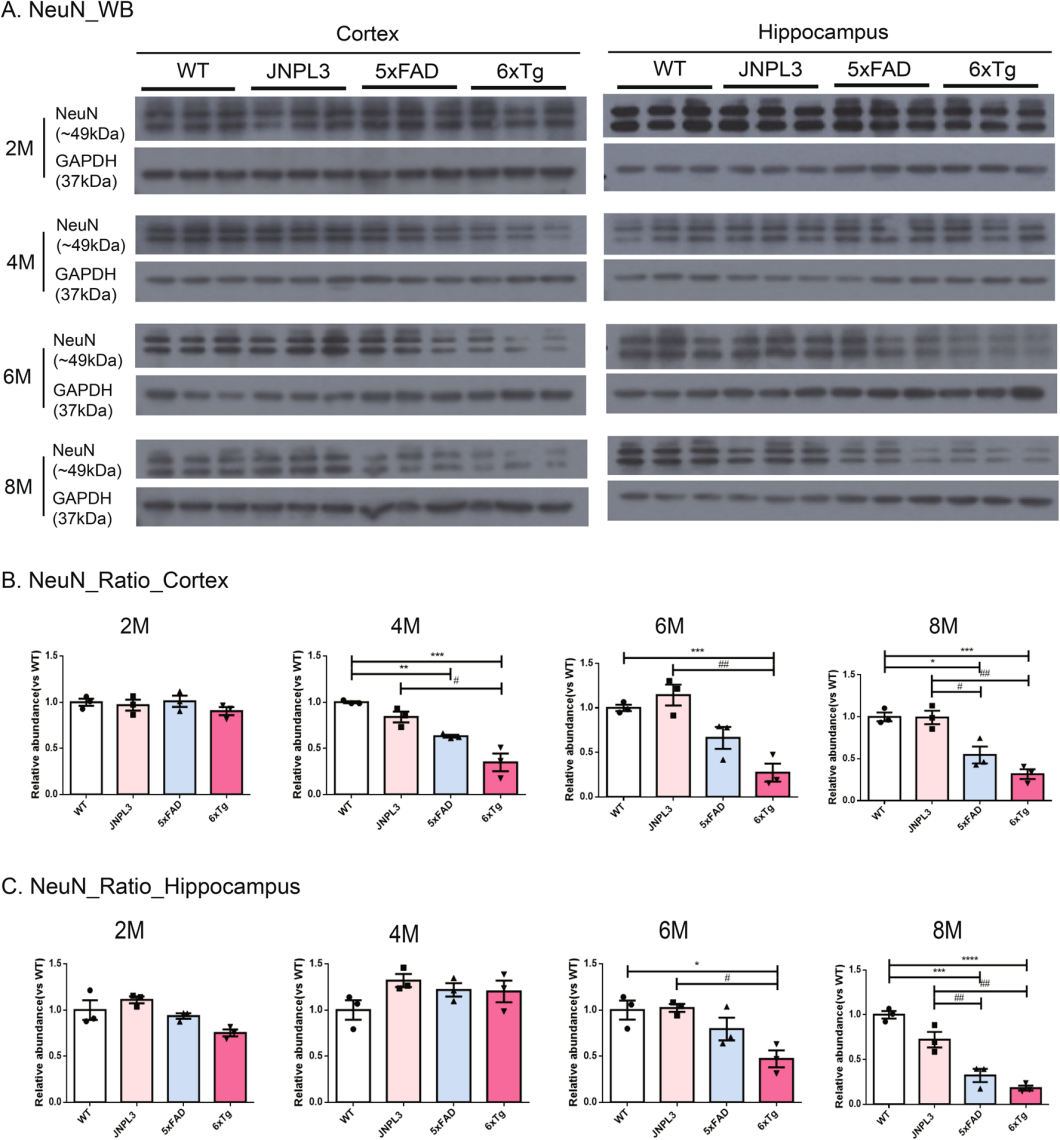
Neuronal cell levels in the cortical and hippocampal area of the 6xTg brain. Neuronal cells (NeuN, 46~48 kDa) were detected in cortical and hippocampal tissue lysates of 2-, 4-, 6-, and 8-month-old WT, JNPL3, 5xFAD, and 6xTg mice by Western blot analysis. (**A**) Representative Western blots of NeuN from cortical and hippocampal brain lysates. In Fig. S5, full-length blots were presented. (**B**) The graph shows the percentage of the density of NeuN normalized to GAPDH on Western blot bands from cortical and hippocampal tissue lysates. B) In the cortex, 4-, 6- and 8-month-old 6xTg mice showed significantly decreased NeuN compared with 5xFAD and WT mice. (**C**) In the hippocampus, 6- and 8-month-old 6xTg mice showed significantly decreased NeuN compared with 5xFAD and WT mice. All data are shown as means ± SEM, and each experiment was repeated five times (n = 3 per group). Statistical analyses were performed by one-way ANOVA by the Tukey's multiple comparisons test. ****p < 0.0001, ***p < 0.001, **p < 0.01 vs. WT; ^####^*p* < 0.0001, ^###^*p* < 0.001, ^##^*p* < 0.01 vs. JNPL3; ^$$$$^*p* < 0.0001, ^$$$^*p* < 0.001 vs. 5xFAD.

Next, we screened the neuronal loss in 2-, 4-, 6-, and 8-month-old WT, 5xFAD, and 6xTg mice. This is because the marked synaptic loss in the brains of 6xTg mice has been identified from 4 months of age, which was earlier than expected. At 2 months of age, neuronal loss was undetectable in all groups of mice (Fig. 6A). In the hippocampus, no significant differences could be detected between JNPL3, 5xFAD, or 6xTg mice at 4 months of age (*p* > 0.05). However, neuronal loss first appeared and significantly decreased in the cortex of 6xTg mice relative to WT, JNPL3, and 5xFAD mice (0.35 ± 0.09, *p* < 0.05 vs. WT) (Fig. 6B). The neuronal loss increased progressively with age in 6xTg mice from 4 to 8 months, but not in WT mice (Fig. 6). In the hippocampus of 6xTg mice, neuronal loss was significantly decreased (0.47 ± 0.09, *p* < 0.05 vs. WT, *p* < 0.05 vs. JNPL3) relative to WT and JNPL3 mice at 6 months of age. This was also the case in the cortex (0.27 ± 0.1, *p* < 0.001 vs. WT, *p* < 0.01 vs. JNPL3) (Fig. 6B,C).

Immunohistochemistry was performed using 6-months old WT, JNPL3, 5xFAD, and 6xTg mice to confirm the western blot results showing neuronal loss and synaptic loss, and the results obtained were similar to the western blot results. The levels of synaptophysin immunoreactivity were significantly reduced in both the cortex and hippocampus of the 6-month-old 6xTg mice compared to age-matched WT and JNPL3 mice (cortex: 838.4 ± 45.34, *p* < 0.01 vs. WT, *p* < 0.05 vs. JNPL3; hippocampus: 782.9 ± 22.34, *p* < 0.01 vs. WT, *p* < 0.01 vs. JNPL3) (Fig. S9). Compared to 5xFAD (cortex, 660.4 ± 33.04; hippocampus, 569.7 ± 236.55), synaptophysin immunoreactivity tended to decrease at 6xTg, but there was no significant difference (Fig. S9B&C). And the NeuN-positive cells were decreased in 6xTg mice at 6 months (Fig. S9). In particular, NeuN-positive cells in the hippocampus of 6xTg mice were significantly reduced compared to WT and JNPL3 mice at 6 months of age (0.32 ± 0.08, *p* < 0.001 vs. WT, *p* < 0.05 vs. JNPL3). There was a significant decrease in the cortex of 6xTg mice compared to WT and JNPL3 mice at 6 months of age (0.64 ± 0.05, *p* < 0.01 vs. WT, *p* < 0.01 vs. JNPL3) (Fig. S9). However, there was a tendency to decrease in NeuN-positive cells at 6xTg compared to 5xFAD (cortex, 605 ± 24.54; hippocampus, 605 ± 24.54), but there was no significant difference (Fig. S9D&E).

## Discussion

AD, the most common form of dementia, is pathologically characterized by two causative factors, namely Aβ plaques and NFTs^25^. Prominent activation of the inflammatory response is observed in the brain, including activation of the microglia and gliosis. Another implicated factor is synaptic dysfunction and neuronal loss.

Although several candidate therapeutics for AD have shown great promise in Tg mice, and, these studies have rarely translated into clinical benefits for human patients. Therefore, an appropriate animal model of AD is required to develop effective treatments for AD and related diseases. The 5xFAD mouse is the most commonly used animal model in AD research^26^. These animals develop Aβ deposition along with cognitive impairment but do not form NFTs. However, the amyloid plaque is not sufficient to convert the tau to a pathological conformation that drives neurodegeneration accompanying cognitive decline and neuronal loss^27^.

Therefore, we developed a new appropriate AD mouse model (6xTg) showing age-dependent development of Aβ plaques and tau deposition in one animal model. In the 6xTg model, expressions of the transgenes (APP and PSEN1) were not altered from the parental line, 5xFAD, and the expression of the transgene (Tau) was shown to increase slightly more than the parental line, JNPL3 (Fig. S1C). In 6xTg mice, amyloid deposits appeared from 2 months, and NFT developed by 4 months (Figs. S1, S3). Also, Aβ deposition was greater in the cortex than it was in the hippocampus of 2-month-old 6xTg mice, as compared with 5xFAD mice (Fig. 2B&C). At tau deposition, the tendency for PHF tau to develop was increased in the cortex and hippocampus of 6xTg mice as compared to JNPL3 mice at 4 months, but there was no significant difference. However, at 6 months of age, there was a significant increase in PHF tau of 6xTg animals as compared to JNPL3 mice (Figs. 3, S3). These results were supported by a previous report that crossing hAPP Tg mice with hTau Tg mice significantly enhanced tau deposition without changing Aβ deposition^12^. In particular, substantial Aβ deposition was detected in the entorhinal cortex, which contains specialized neurons called grid cells that form part of the spatial navigation system^20^. Previous many studies reported the correlation of subicular damage with severity of AD symptoms^28–30^, and we compared the subiculum regions of all groups using Thio-S staining and found that Aβ deposition appeared only in the 5xFAD and 6xTg mice at 2 months old, and the amount of Aβ deposition had aggressively increased by 4 months (Fig. S8A). Interestingly, there was no difference in 6E10-positive plaques between 5xFAD and 6xTg at 2 months, but Thio-S stained plaques were significantly increased in 6xTg compared with 5xFAD mice.

Besides, Aβ deposition is closely associated with cognitive impairment^2,31^. Cognitive decline is the major clinical manifestation of AD. To confirm whether this new animal model is suitable for preclinical studies, we first verified the cognitive impairment of the 6xTg animal model. Behavioral tests were performed using the Y-maze and MWM, and cognitive impairment was observed from 2 months of age, earlier than it is observed in the 5xFAD model (Fig. 1A,C). These results indicate that 6xTg mice exacerbated behavioral deficits compared to WT and parental lines, JNPL3 and 5xFAD mice.

AD-related brain inflammation is well known to be elevated and is believed to play a distinct role in disease pathogenesis^2^. Also, the presence of Aβ plaques provides stimulation to surrounding glial cells, leading to local immune responses in the human AD brain^32,33^. Microglia activation reflects neuroinflammation in the brain. Therefore, we explored whether the microglia were activated in the brains of 6xTg mice by immunostaining for Iba-1 and MHC class II, which has been reported as an effective marker of activated microglia^34^. Microglial activation occurred in the cortex and hippocampus of 6xTg mice from 2 months and the expression of Iba-1 was significantly increased in the brain of 6xTg mice as compared to WT, JNPL3, and 5xFAD mice (Fig. 4A,B&C). In addition, MHC class II-positive activated microglia were significantly increased in the cortex and hippocampus of 2-month-old 6xTg mice compared with WT, JNPL3, and 5xFAD mice (Fig. S7B&D). These pathological features were sufficient to cause neuronal loss with ensuing cognitive decline.

In this study, synaptic loss and neuronal loss were observed at 6 months of age in 5xFAD, and not observed in JNPL3 until 8 months. Previous reports showed that 5xFAD showed a neuronal loss from 6 to 12 months age^15,35,36^. Recent studies showed that synaptic degeneration and significant neuronal loss of 5xFAD appeared at a slightly faster time point, 4.5—6.5 months of age^19,37^. In 5xFAD mice crossbreed with Tau transgenic lines, there have been various reports for the age at which the synaptic loss and neuronal loss begins to be obeserved^13,15,16^. 5xFAD/PS19 mice have been previously demonstrated a reduced number of CA1 neurons at 9 months of age^13^. In 5xFADxTg30 mice, the number of pyramidal neurons was significantly decreased at 9 months of age^15^. Another study showed that the APP/PS1/rTg21221 line made by crossing APP/PS1 mice with rTg21221 mice overexpress WT human tau showed that addition of human wild‐type tau exacerbates plaque pathology and neurite deformation, but not plaque‐related synaptic loss^16^. The reason seems to be that the time point of observing the pathological phenomenon of the model mouse was limited to a point early or late, not a wide time window. Here, we looked closely at the pathological characters of mice in two-month intervals from 2 to 8 months of age. Synaptic loss and neuronal loss were observed with reduced levels of synaptophysin and NeuN protein expression, respectively, by western blot analysis. Interestingly, loss of neurons was observed at an earlier age in 6xTg mice compared with the previous study. In our 6xTg model mice, the synaptic loss was observed in the cortex from 4 months of age and in the hippocampus from 6 months of age (Fig. 5), and the neuronal loss appeared in the cortex from 4 months of age and in the hippocampus from 6 months of age (Fig. 6), earlier than it is observed in the 5xFAD and JNPL3 models. These results were reconfirmed using IHC, and the results obtained from IHC also confirmed a decrease in the levels of synapses or neurons in the brains of 6xTg compared to WT or JNPL3 (Fig. S9). However, the Western blot quantification presented in Fig. 5C indicates a statistically significant difference in the Synaptophysin ratio in the hippocampus between 5xFAD and 6xTg mice at 6 months. In contrast, the IHC quantification shown in Fig. S9C does not indicate a difference in Synapthophysin between 5xFAD and 6xTg. This discrepancy between WB and IHC results may be understood by experimental methods, even with the same antibody. Usually, the western blot is believed to be more accurate for quantifying the expression level of specific proteins in tissue homogenates or extracted samples than IHC because we can detect a clear specific band on the blot at the expected size. Early formation of the neuritic plaque and tau deposition could further accelerate synaptic reduction and neuronal loss associated with neuropathological changes in AD^2^.

The limitations of this study don’t include the study of the characterization of 6xTg female mice. Also, several questions related to Aβ plaques and NFT, such as the exact triggers of Aβ plaques and NFT formation, the association between plaques and NFT, and how these factors contribute to AD symptoms, remain poorly understood. If additional experiments are carried out to find the answer to these questions utilizing the animal model we developed, we can improve our understanding of AD pathophysiology.

In conclusion, this study reveals that the 6xTg mouse is a promising new AD animal model that presents amyloid and tau pathology earlier than existing AD animal models and may be suitable for preclinical treatment studies of AD.

## Supplementary Information

Supplementary Information.
