# Supplementary material for: Spatial memory deficiency early in 6xTg Alzheimer’s disease mouse model
Source: Sci Rep. 2021 Jan 14;11:1334. doi: 10.1038/s41598-020-79344-5 (PMC7809274; doi:10.1038/s41598-020-79344-5)
Supplement: Supplementary file 1 — Supplementary Information. [file 41598_2020_79344_MOESM1_ESM.pdf]

Spatial memory deficiency early in 6xTg Alzheimer's disease mouse model

ShinWoo Kang[1,2], Jinho Kim[2,3], Keun-A Chang[1,2,3*]

[1]Department of Pharmacology, College of Medicine, Gachon University, Incheon, Republic of Korea

[2]Neuroscience Research Institute, Gachon University, Incheon, Republic of Korea

[3]Department of Health Sciences and Technology, GAIHST, Gachon University, Incheon, Republic of Korea

[*]**Correspondence:** Keun-A Chang, Ph.D., Department of Pharmacology, College of Medicine, Gachon University, 155 Gaetbeol-ro, Yeonsu-gu, Incheon 21999, Korea, E-mail: keuna705@gachon.ac.kr; Tel: +82-32-899-6411

Materials and Methods

Immunohistochemistry

For immunohistochemistry on frozen sections, the 30 μm-thick brain sections were washed three times in PBS containing 0.2% Triton X-100 and were then incubated in a blocking solution (0.5% bovine serum albumin and 3% normal goat serum in PBS with 0.4% Tween 20) for 1 h at RT[21]. For MHC class II/Thio-S double staining, the sections were incubated with the primary MHC Class II (Novus Biologicals, Littleton, CO, USA) overnight at 4°C. Following this, the sections were washed three times and incubated in filtered 1% aqueous Thioflavin-S for 10 minutes at RT. After washing, an Alexa Fluor 555 Donkey anti-mouse IgG antibody (Invitrogen) was incubated for 1 hour at RT. For NeuN/Synaptophysin double immunofluorescence staining on frozen sections, the sections were double-stained with NeuN (Millipore, Massachusetts, USA 1:500) and Synaptophysin (Abcam, Cambridge, UK 1:100) overnight at 4°C. After washing, Goat-anti-Rabbit IgG Alexa Fluor 488 (Invitrogen, California, USA) and Goat anti-Mouse IgG Alexa Fluor 555 (Invitrogen) were stained together for 1 hour at RT.

Next, the brain sections were then washed three times and mounted onto slides using Antifade Mounting Medium with DAPI (Vector Laboratories, Burlingame, CA, USA). Specimens were taken using a Nikon TS2-S-SM microscope (Nikon Microscopy, Tokyo, Japan) equipped with a Nikon DS-Qi2 camera. For quantification of IHC, the number of Aβ plaques was quantified by blind counting and indicated as numbers in the defined area (5 mice per group). The coverage of MHC II-, synaptophysin-, or NeuN-positive cells per region were measured in each ROI region and analyzed using NIS-Elements software (BR 4.40.00, Nikon Microscopy). Once the ROIs were defined, the green channel showing Alexa Fluro 488, and the red channel showing Alexa Fluro 555 was used to measure the intensity of green or red signal within each ROI per section[23].

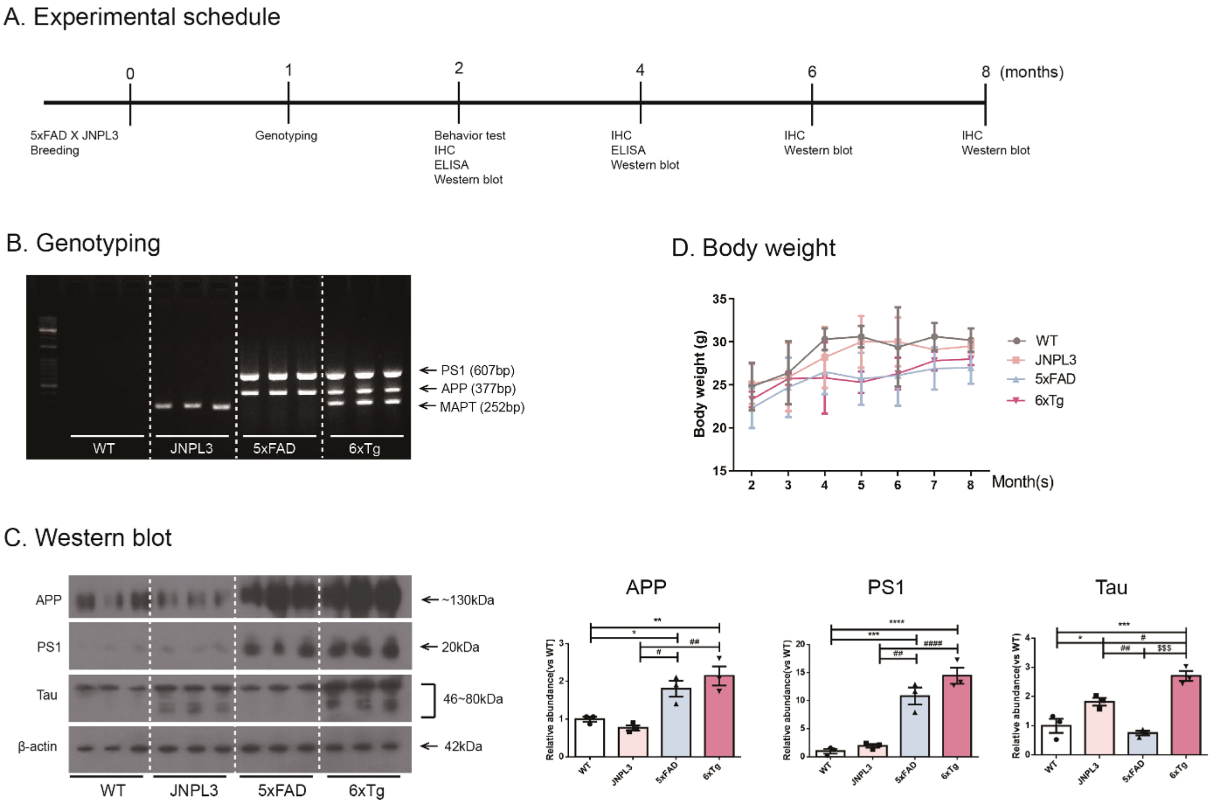

Fig. S1. The experimental scheme, genotyping, and body weight.

(A) Experimental schedule by age (months) (B) DNA generated using mice ear tissues were genotyped by PCR analysis. PCR genotyping analysis demonstrates the APP (377 bp)/PS1 (607 bp) mRNA expression in the brains of 5xFAD mice, MAPT (252 bp) mRNA expression in the brains of JNPL3 mice, and APP (377 bp)/PS1 (607 bp)/MAPT (252 bp) mRNA expression in the brains of 6xTg mice. (C) Representative western blots of APP (~130kDa), PS1(20kDa), and Tau (H150) (46~80kDa) from cortical brain lysates. Full-length blots are presented in Supplementary Figure 6. The graph shows the percentage of protein density normalized for β-actin in the western blot band obtained using antibodies such as PSEN1, 22C11, and Tau (H150) from cortical tissue lysate (n = 3 per group). (D) Body weights of WT, JNPL3, 5xFAD, and 6xTg mice by age (months). All data are given as means ± SEM (n = 8–20 per group). Statistical analyses were performed by one-way ANOVA followed by Tukey's multiple comparisons test. **** $p < 0.0001$, *** $p < 0.001$, ** $p < 0.01$ vs, * $p < 0.05$ vs. WT; #### $p < 0.0001$, ## $p < 0.01$, # $p < 0.05$ vs. JNPL3; $$$ $p < 0.001$, vs. 5xFAD.

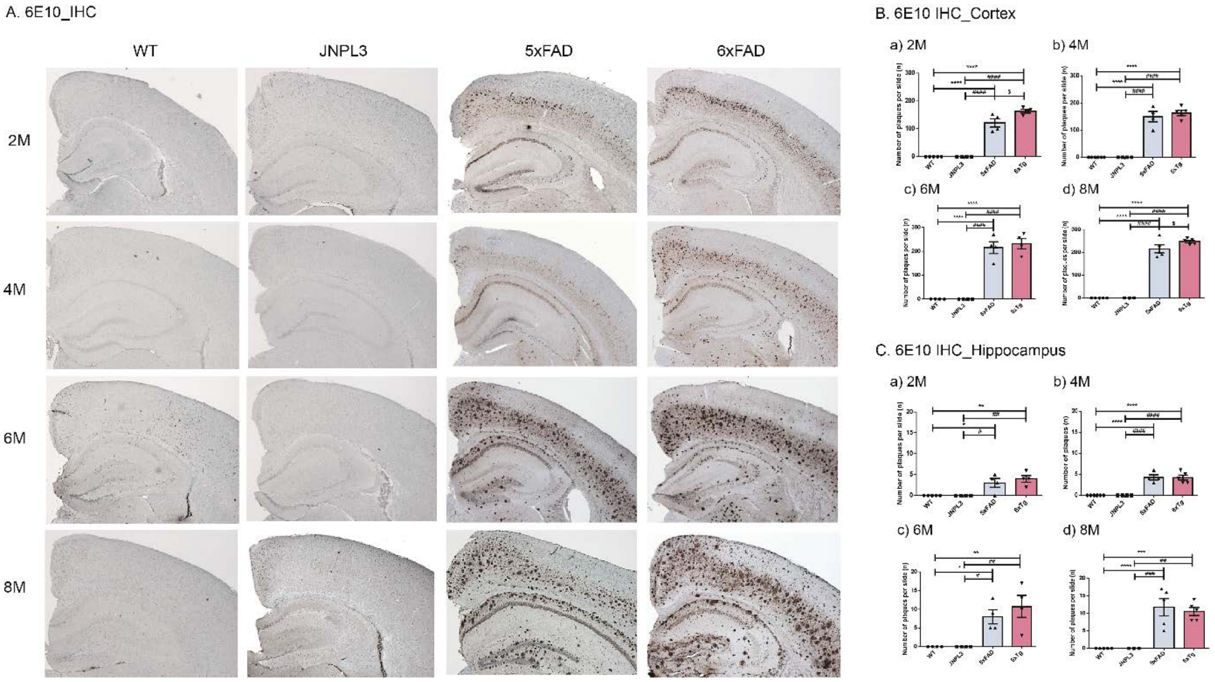

Fig. S2. Aβ plaques were identified in the brains of WT, JNPL3, 5xFAD, and 6xTg mice by immunohistochemistry.

(A) Immunostained brain tissues with 6E10 antibody of 2-, 4-, 6-, and 8-month-old 6xTg mice and their age- and gender-matched WT, JNPL3, 5xFAD, and 6xTg counterparts (n = 3–7 mice per group). (B–C) In the 2-, 4-, 6-, and 8-month-old mice, Aß deposition in the cortex and hippocampus of 6xTg mice showed no significant difference compared the 5xFAD mice in the cortex, but showed a tendency to decrease. All data are given as means ± SEM. Statistical analyses were performed by one-way ANOVA followed by Tukey's multiple comparisons test. **** $p < 0.0001$, *** $p < 0.001$, ** $p < 0.01$ vs. WT; #### $p < 0.0001$, ### $p < 0.001$, ## $p < 0.01$ vs. JNPL3; $$ $p < 0.01$, $ $p < 0.05$ vs. 5xFAD.

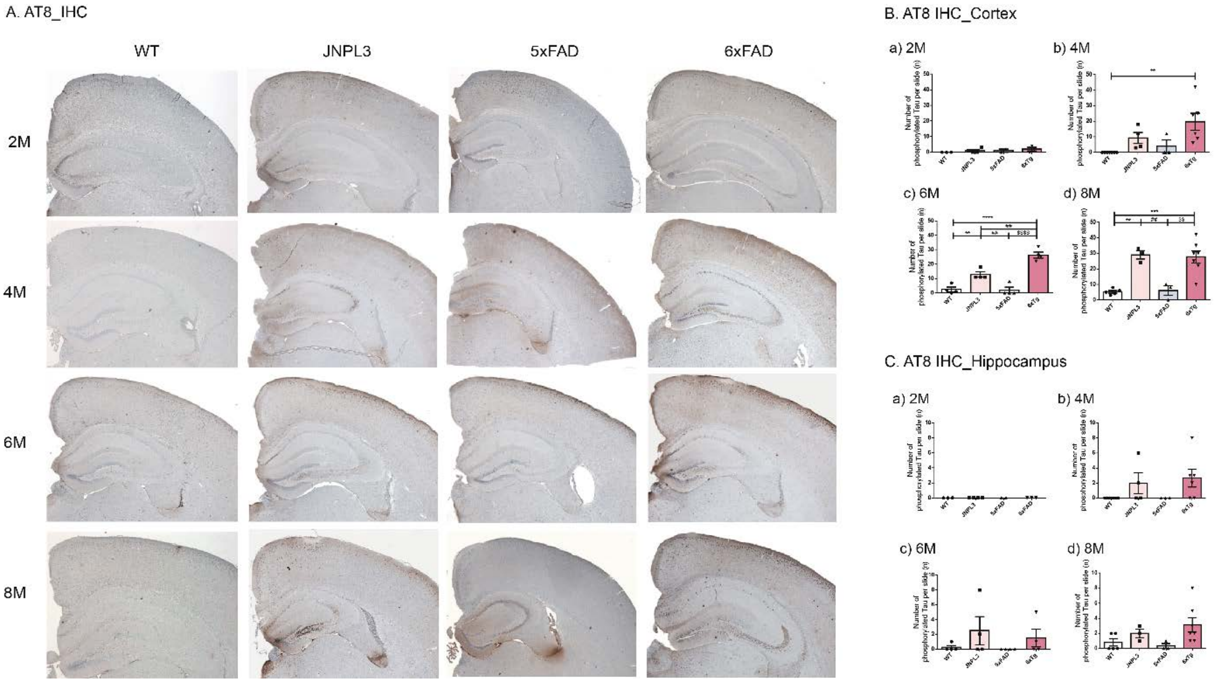

Fig. S3. PHF tau deposits were identified in WT, JNPL3, 5xFAD, and 6xTg mice by immunohistochemistry.

(A) Immunostained brain tissues with AT8 antibody of 2-, 4-, 6-, and 8-month-old 6xTg mice and their age- and gender-matched WT, JNPL3, 5xFAD, and 6xTg counterparts (n = 3–7 mice per group). (B–C) In the 4-, 6-, and 8-month-old mice, PHF tau in the brains of 6xTg mice significantly increased compared with WT mice, but there was no significant difference compared with the JNPL3 mice in the cortex or hippocampus, but there was a tendency to decrease. All data were given as means ± SEM. The statistical analyses were performed by one-way ANOVA followed by Tukey's multiple comparisons test. **** $p < 0.0001$, *** $p < 0.001$, * $p < 0.05$ vs. WT; ### $p < 0.001$, ## $p < 0.01$ vs. JNPL3; $$$$ $p < 0.0001$, $$$ $p < 0.001$, $ $p < 0.05$ vs. 5xFAD.

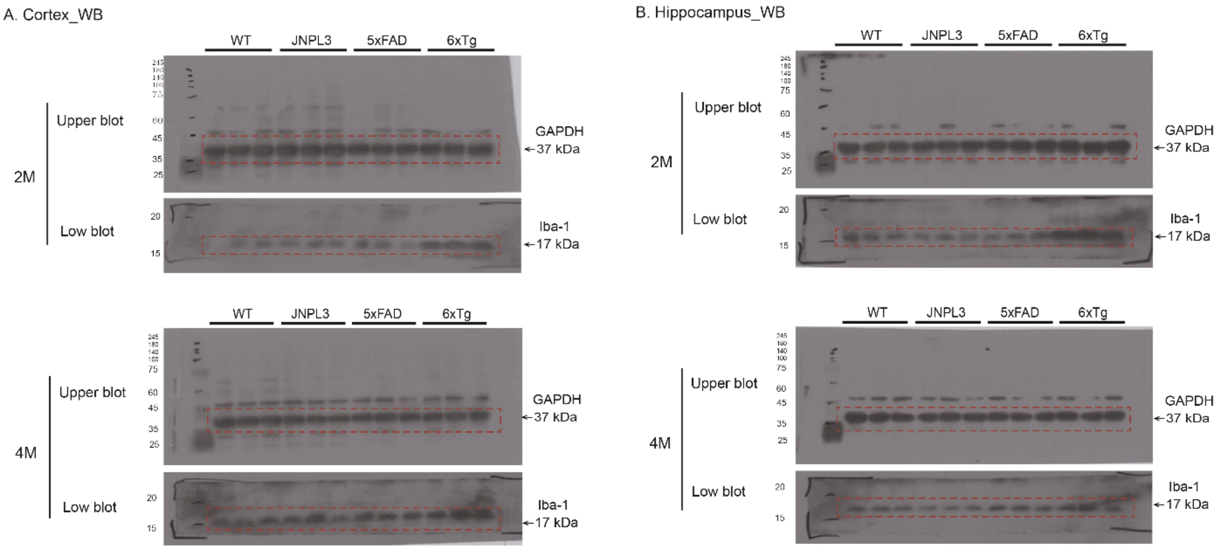

Fig. S4. The original western full blot.

Western blot analysis of cortical (A) and hippocampal (B) extracts from 2- and 4-month-old mouse brain tissues was performed using antibodies against Iba-1 (NBP2-19019) (lower) and GAPDH (A531) (upper). The full blot (15~245kDa) was cut at a position of 25 kDa and divided into two blots to expose to different target antibodies. The cropped images to the part indicated by the red dotted box of these full blots were shown in figure 4.

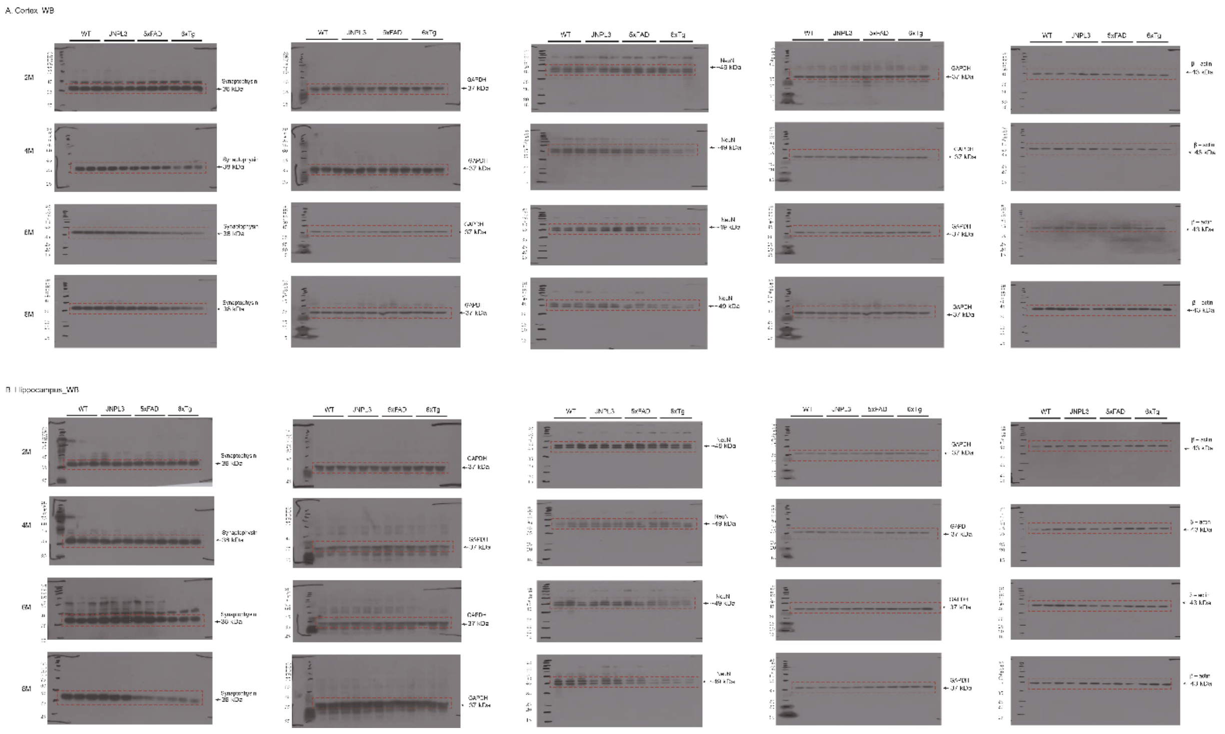

Fig. S5. The original western full blot.

Western blot analysis of cortical (A) and hippocampal (B) extracts from 2-, 4-, 6-, and 8-month-old mouse brain tissues was performed using antibodies against NeuN (ABN78), Synaptophysin (ab8049), GAPDH (A531), and β-actin (SC4777/8). The cropped images to the part indicated by the red dotted box of these full blots were shown in figure 5 and figure 6.

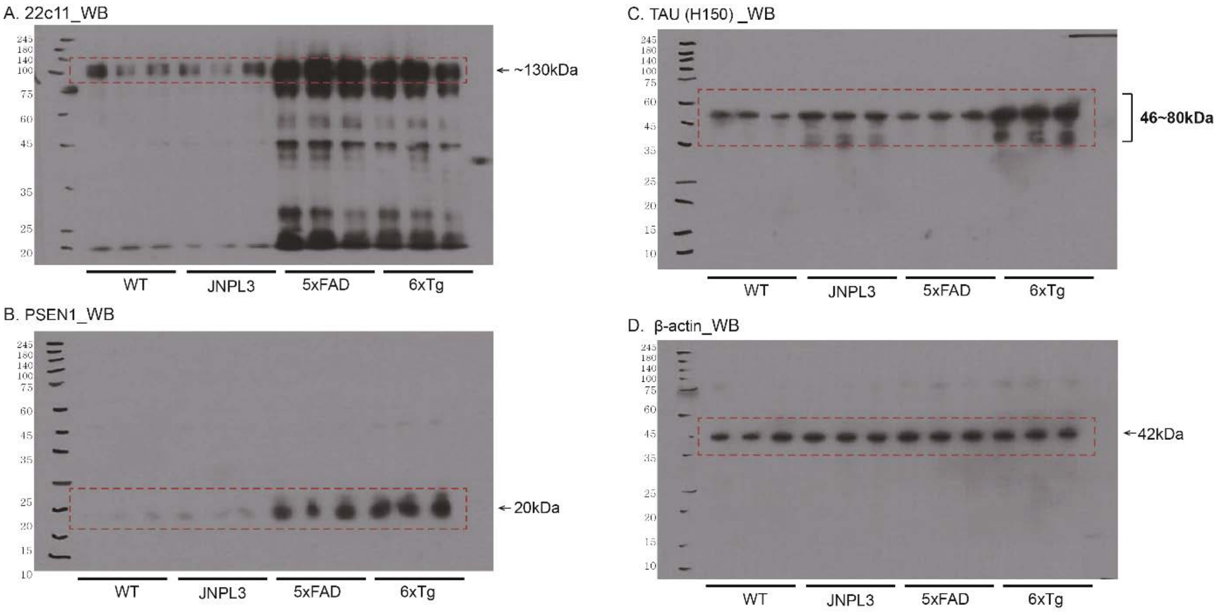

Fig. S6. The original western full blot.

Western blot analysis of extracts from mouse brain tissues was performed using APP (22c11, MAB348), PS1 (CS5643), Tau (H-150, SC5587), and β-actin (sc47778). Representative full blots are shown.

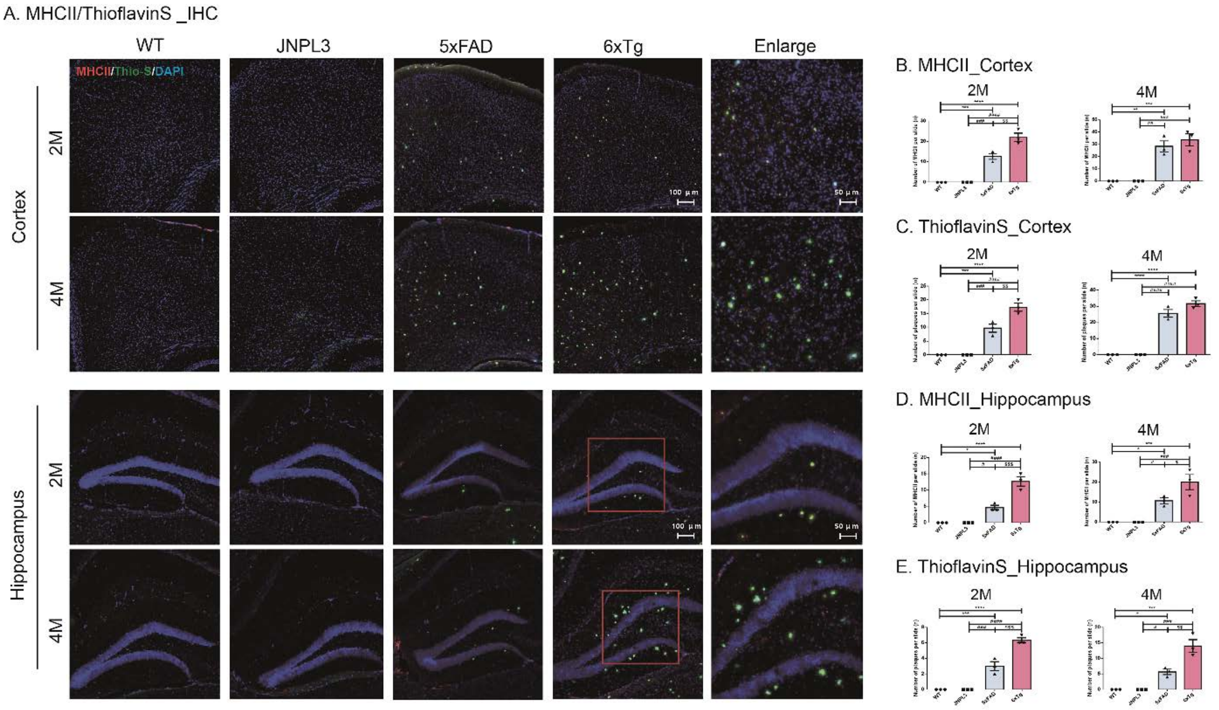

Fig. S7. Amount of Aβ plaques and activated microglia in the cortex and hippocampus of WT, JNPL3, 5xFAD, and 6xTg mice brains.

(A) Immunohistological staining of amyloid plaques with thioflavin-S (green) and anti-MHC class II antibody (red) in the cortex and hippocampus of 2- and 4-month-old WT, JNPL3, 5xFAD, and 6xTg mice by immunofluorescent staining. (B) Quantification analysis of thioflavin-S stained amyloid plaques and MHC class II-positive cells in the cortex and hippocampus of 2- and 4-month-old WT, JNPL3, 5xFAD, and 6xTg mice were represented as a number of plaques per slide (n) and a number of MHC class II per slide (n). All data were given as means ± SEM. The statistical analyses were performed by one-way ANOVA followed by Tukey's multiple comparisons test. **** $p < 0.0001$, *** $p < 0.001$, ** $p < 0.01$, * $p < 0.05$ vs. WT; #### $p < 0.0001$, ### $p < 0.001$, ## $p < 0.01$, # $p < 0.05$ vs. JNPL3; $$$ $p < 0.001$, $$ $p < 0.01$, $ $p < 0.05$ vs. 5xFAD.

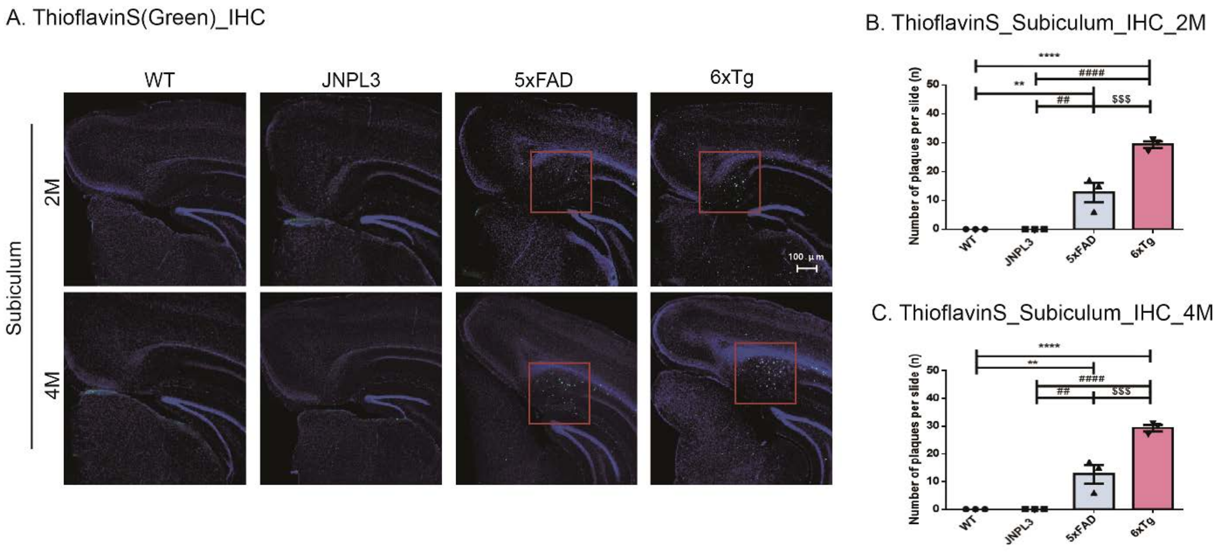

Fig. S8. Amount of Aβ plaques in the subiculum of WT, JNPL3, 5xFAD, and 6xTg mice brains. (A) Immunohistological staining of amyloid plaques with thioflavin-S in the subiculum of 2- and 4-month-old WT, JNPL3, 5xFAD, and 6xTg mice by staining. (B) Quantification analysis of thioflavin-S stained amyloid plaques in the subiculum of 2- and 4-month-old all group mice were represented as a number of plaques per slide (n). All data were given as means ± SEM. The statistical analyses were performed by one-way ANOVA followed by Tukey's multiple comparisons test. **** $p < 0.0001$, ** $p < 0.01$ vs. WT; #### $p < 0.0001$, ## $p < 0.01$ vs. JNPL3; $$$ $p < 0.0001$ vs. 5xFAD.

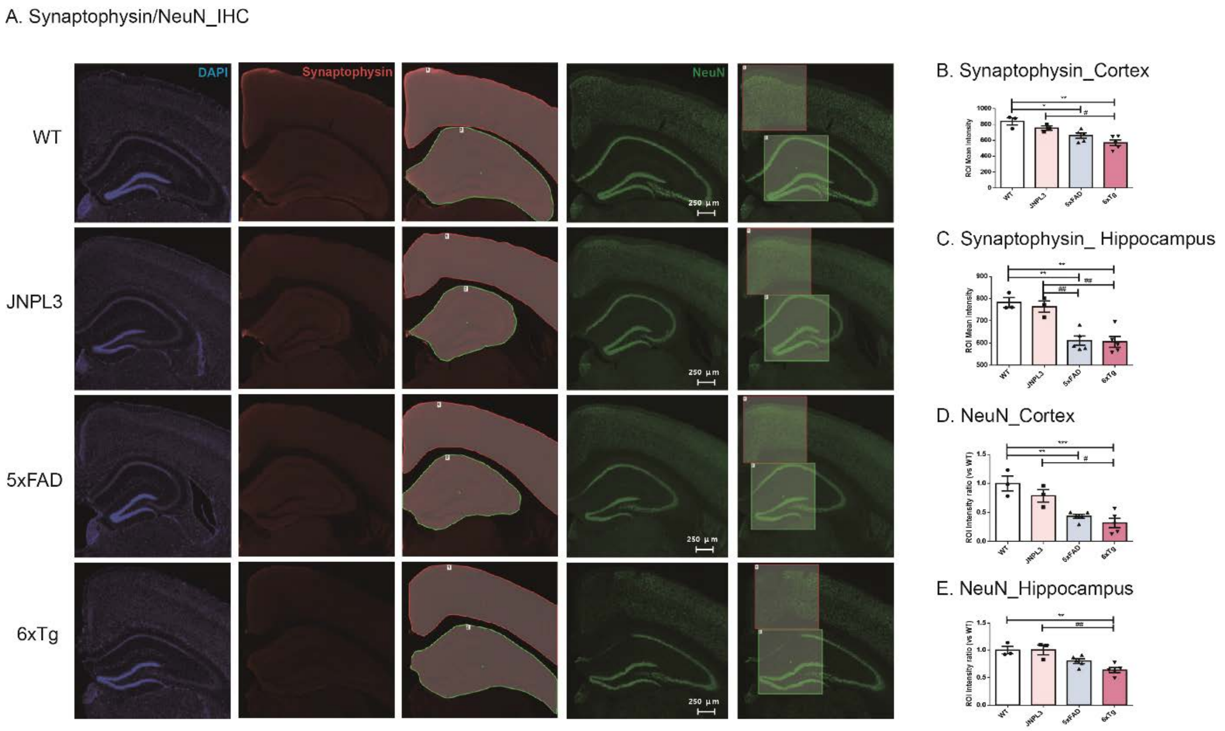

Fig. S9. NeuN and Synaptophysin cell type expressed in the Cortex and Hippocampus of WT, JNPL3, 5xFAD, and 6xTg mice brains.

(A) Representative synaptophysin (red)- and NeuN (green)-immunofluorescent images in the brains of 6-month-old WT, JNPL3, 5xFAD, and 6xTg mice. (B) Quantification analysis of synaptophysin and NeuN-positive cells in the cortex and hippocampus of 6-month-old WT and 6xTg mice were represented as an ROI mean intensity. Red ROI No.1 represents the cortical region and green ROI No.2 represents the hippocampal region. All data were given as means ± SEM. The statistical analyses were performed by one-way ANOVA followed by Tukey's multiple comparisons test. *** $p < 0.001$, ** $p < 0.01$, * $p < 0.05$ vs. WT; ## $p < 0.01$, # $p < 0.05$ vs. JNPL3.
